# eNOS Gene Variant in Patients with Coronary Artery Disease

**DOI:** 10.1155/2013/403783

**Published:** 2012-11-28

**Authors:** Milad Abolhalaj, Mahsa M. Amoli, Parvin Amiri

**Affiliations:** Endocrinology and Metabolism Research Center (EMRC), Tehran University of Medical Sciences, Tehran 14114, Iran

## Abstract

*Subject & Aim*. Endothelial nitric oxide synthase (eNOS) is one of the most important candidate genes in CAD. A functional polymorphism within eNOS gene is a 27 bp VNTR on its intron 4 which has been shown to be associated with various diseases. In this study we investigated eNOS VNTR polymorphism in addition to eNOS gene expression profile in patients with CAD. *Material and Methods*. The study comprised patients with angiographically confirmed CAD (CAD^+^) and individuals with normal coronary as CAD^−^. eNOS VNTR polymorphism frequencies were determined in both groups. In addition eNOS gene expression profile was examined using a quantitative real-time PCR. *Results*. We have found that aa genotype was significantly increasing the risk of CAD in our patients (aa versus ab + bb, *P* = 0.02, OR = 3.5; 95% CI: = 0.98 to 16.2). The differences in eNOS expression were not significant between patients and normal group; however in CAD^+^ patients eNOS expression was higher than the expression level of patients carrying other genotypes (*P* = 0.16). *Conclusion*. We have observed that eNOS gene polymorphism was associated with CAD in angiography-confirmed patients. However, the difference in eNOS gene expression was not statistically significant between patients and control which might be due to the contribution of other confounding factors which require further investigations.

## 1. Introduction

 Coronary artery disease (CAD) is one of the leading causes of death in the world. Familial aggregation of CAD indicates the contribution of genetic factors which might be involved in disease development. Genome-wide association studies have identified approximately 34 distinct loci in correlation with CAD [1]. Nitric oxide synthase (NOS) is one of the most important candidate genes in CAD. It is synthesizing NO in a catabolic reaction in presence of L-arginine [2]. The gene is located on chromosome 7q36 and is comprised of 3 isoforms in mammalian cells: neuronal (nNOS, type I), inducible (iNOS, type II), and endothelial (eNOS, type III). Most circulating NO is produced by these three isoforms [3, 4]. In all isoforms calmodulin domains become activated in the presence of Ca^+2^ resulting in enzyme activation [5]. Additionally, in the presence of stress, phosphorylations at Ser1177 of eNOS lead to Ca^+2^ sensitiveness which in turn results in enzyme activation [6, 7]. 

 Vascular endothelial growth factor (VEGF) can also give rise to eNOS enzyme activation due to Ser/Thr kinase (Akt) function [8]. NO has essential role in vasodilatation via soluble guanylyl cyclase and cyclic GMP generation in smooth muscle cells [9–11]. Likewise, NO can inhibit leucocyte adhesion to the vessel wall by either interfering with the ability of the leucocyte adhesion molecule CD11/CD18 to bind to the endothelial cell surface or by suppressing CD11/CD18 expression on leucocytes [12–15] resulting in atheroma formation which in turn contributes to the formation of a soft plaque which increases the risk of unstable angina, thrombosis, and acute myocardial infarction [16].

 eNOS-derived NO also contributes to the prevention of apoptosis in endothelial cells by reactive oxygen species (ROS) and Angiotensin II (AT) [17]. Furthermore, in presence of NO, smooth muscle cell proliferation is inhibited through the prohibition of DNA synthesis [18, 19]. Likewise, endothelium-derived NO acts as potent regulator of blood pressure and blood flow [20]. Increased level of iron could lead to free hydroxy radicals production resulting in LDL oxidation [21] which is specified as a key factor in pathogenesis of atherosclerosis and cardiovascular diseases [22] due to a lipid accumulation in macrophages and foam cells, which is toxic for cells [23]. Moreover, NO's protective effects on animal endothelium have also been demonstrated in mice with impaired eNOS function giving rise to hypertension [24] which has a close correlation with CAD [25]. Previous studies have reported eNOS polymorphisms as probable risk factors in the CAD pathogenesis [26]. A functional polymorphism within eNOS gene is a 27 bp VNTR on its intron 4, which is comprised of 2 alleles (4a, 4b), and has been studied in several conditions [27]. Various frequencies for this polymorphism have been reported in different ethnic groups [27, 28]; in this study we investigated eNOS 27 bp VNTR polymorphism in addition to eNOS gene expression profile in Iranian CAD^+^ people in comparison with CAD^−^ patients.

## 2. Materials and Methods

DNA was extracted from anticoagulated blood collected in EDTA tubes using salting-out method. Genotyping was carried out as follows: A total of 20 ng genomic DNA was amplified in a 10 *μ*L final PCR reaction volume containing 5 pmol of each primers: forward primer 5′-GGG AACCTCAGCCCAGTAGTGAA-3′ and reverse primers 5′-TCTCTTAGTGCTGTGGTCAC-3′, 200 mmol of dNTPs, 10 uL NH_4_ buffer, and 0.6 units of Taq polymerase. The DNA was denatured at 95°C for 2 min, and temperature cycling was set at 95°C for 45 s, 58°C for 45 s, and 72°C for 45 s for 40 cycles followed by a final extension at 72°C for 5 min. PCR product was visualized on a 4% agarose gel stained with ethidium bromide.

Gene expression analysis was performed in fresh un-stimulated peripheral blood mononuclear cells (PBMCs) separated from 5 cc heparinized blood sample collected form each patient. After density gradient lymphocyte separation using ficoll (1.077), the total RNA was extracted using TriPure Isolation Reagent (Roche Applied Science), according to the manufacturer's protocol. 1 *μ*g aliquot of total RNA from each sample was reverse transcribed into single-stranded cDNA using random hexanucleotides primers and expanded reverse transcriptase (Roche Applied Science). cDNA for each sample was subjected to quantitative real-time PCR for internal quantitative control and eNOS (Table 2). All quantitative PCR reactions were performed in mixtures containing cDNA, 10 mL RT2 Real-Time SYBR Green/ROX PCR Master, primer pairs, and nuclease-free water. Each biological replicate was run in duplicate on an ABI step one quantitative PCR system. 

## 3. Statistical Analysis

 Our gene expression data were normalized against HPRT as our reference gene. Data analysis was conducted using, Livak formula, the 2^−ΔΔC_T_^ method [29]. The significance of differences in gene expression between control and test groups was established by Student's *t*-test considering the fact that our data on eNOS gene fold changes had a normal distribution. In addition, regarding nominal nature of VNTR variables we used crosstabs benefiting from Chi-square test to show significant differences related to our target genotype, a, versus two other genotypes, ab + bb. In addition, using ANOVA and error bars we attempted to investigate the possible changes in eNOS gene expression levels in the presence of individual genotypes. Analysis was performed by SPSS version 16, and *P* ≤ 0.05 was considered as significant.

## 4. Results

**Figure 1:**
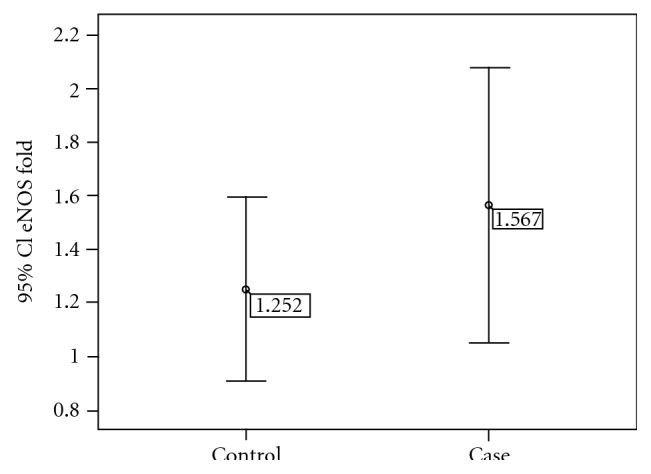
eNOS expression in patients with CAD^+^ versus CAD^−^ Error-Bar shows mean ± SD mRNA expression normalized against HPRT internal control in patients with CAD (CAD^+^) and patients without CAD (CAD^−^). There was a nonsignificant increase in eNOS expression in CAD^+^ group versus CAD^−^ (*P* = 0.29).

**Figure 2:**
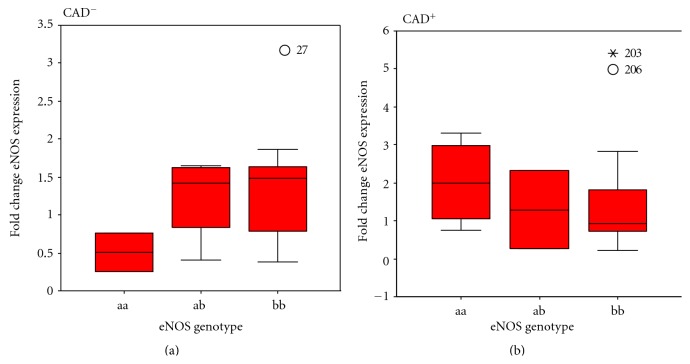
Enos gene expression in patients caryying different intron 4 VNTR genotypes. Box plots shows mRNA expression in (a) controls (CAD^−^) and (b) cases (CAD^+^) stratified based on VNTR polymorphism with aa, bb, and ab genotypes. There was a decrease in expression of eNOS in patients carrying aa genotype in CAD^−^ group and an increase in eNOS expression in CAD^+^ patients. The differences were not significant *P* > 0.05.

The characteristics of patients included in this study are shown in Table 1. We discovered that frequency of patients carrying aa genotype was significantly higher in the CAD^+^ group compared to CAD^−^ group (*P* = 0.02, OR = 3.5; 95% CI: = 0.98 to 16.2) (Table 3). Quantitative real-time PCR analysis demonstrated that eNOS expression was upregulated in the CAD^+^ group in comparison with control people (Figure 1); however, in spite of the fact that an upregulated expression was detected, it was not statistically significant in the 0.05 significance level (*P* = 0.29). Additionally, we found that in CAD^−^ patients carrying aa genotype eNOS expression was lower than patients with ab or bb genotypes. This was not observed in CAD^+^ group (Figure 2). The differences were not singnificant (*P* > 0.05).

## 5. Discussion

 All NOS gene's isoforms are present in atherosclerosis although there is a powerful evidence pointing out to eNOS-defensive effects on vessels' wall against atherosclerosis [30]. Due to eNOS-protective roles and its contribution to a variety of events in cardiovascular diseases, this gene and its functional polymorphism have attracted great attention [31–33]. 

Controversial results have been reported with respect to eNOS expression in patients with CAD. In a report by De Belder and colleagues they have demonstrated a decrease in both eNOS activity and expression [34], while other studies have shown an increase in the eNOS activity and expression in CAD patients which is regarded as a compensatory mechanism against CAD [35, 36]. We have found a nonsignificant increase of eNOS gene expression in unstimulated PBMCs of patients with CAD. We also investigated the association between intron 4, 27 bp VNTR polymorphism and CAD, and we observed that it was significantly associated with CAD in Iranian subjects. Our data shows that carriers of aa genotype had increased risk of CAD. Similarly, Matyar et al., have shown that aa genotype frequency was significantly higher in CAD people in southern Turkey [37]. In addition, an association between this polymorphism and CAD in smokers with severely constricted arteries has been reported by Wang et al. [38]. In another study eNOS 4a allele was considered as an independent risk factor for myocardial infarction (MI) with no considerable difference between smokers and nonsmokers in Japanese population [39]. Likewise, Kunnas et al., have related the polymorphism to both CAD risk and MI in the Finish middle-aged men [40]. Similarly, there was a relationship between CAD and acute coronary syndrome (ACS) and intron 4 VNTR polymorphism in African-American, Caucasian men, and Koreans [41, 42]. However, other groups have failed to detect a significant difference between CAD and this polymorphism in German and Taiwanese population [43–45].

In addition eNOS polymorphism was not associated with acute myocardial infarction(AMI) and coronary heart disease (CHD) in southern Indian [36] and Greek patients [46]. In a meta-analysis by Casas et al., it was shown that 9 out of the 16 included studies found an increased risk for ischemic heart disease (IHD) in individuals homozygous for the a allele compared with b allele carriers (b/b plus b/a), but only in one study the difference was statistically significant [33].

 The reason for such a discrepancy remains ambiguous. A great number of factors seem to be involved including diagnostic criteria or difference in studied ethnic population. For instance, the frequency of the aa genotype was nearly 25% in our subjects which was similar to previously reported results in African-Americans (26 to 30%) [40, 41], Taiwanese (23.7%) [45], and south Indian population (24%) [36]. This was higher than reported frequencies for Spanish (13%), Turkish (14%), Japanese (14%), Caucasian of Australia (17%), and Koreans (19%) [36].

 Although our results showed no significant difference in eNOS expression among individual genotypes, CAD patients had approximately 4 times higher rate of eNOS expression in the presence of a allele (aa genotype) in comparison with the same genotype in normal group. 

 Tsukada et al., have found that a strong association between eNOS VNTR and nitric oxide plasma level and mean plasma nitric oxide level in their subjects with aa genotype was about 20% lower than subjects with bb genotype [47]. Interestingly, an almost similar pattern was observed in our CAD^−^ group, but not in CAD^+^ group. With respect to this issue, Yoon and colleagues have demonstrated that there was a fundamental effect of eNOS4a/b polymorphism on variance of plasma nitric oxide concentrations in Korean population which was dependent on other factors including smoking [32]. The independent association between this polymorphism and plasma NO concentration was not found by other studies [48]. It has been suggested that the effect of this polymorphism on eNOS gene expression might be due to its contribution to exon splicing. Recent data represents that the polymorphism is likely to be source of a 27nt-long RNA derived from pre-mRNA splicing which is able to suppress eNOS expression and may introduce a new class of small RNA [49, 50]. It was shown that a allele in given doses [51] has the capability to produce lower levels of 27nt-small RNA and consequently higher levels of eNOS mRNA compared with cells carrying b allele [50]. It has been shown that such increase in eNOS expression in presence of aa genotype has a strong association with survival in advanced stage of nonsmall cell lung cancer [52].

Our study was conducted on small number of samples which was one of the main limitations that necessitate careful interpretation of results. Considering the low frequency of a allele further studies on larger population would certainly be more conclusive. However, our data confirms the previous reports, which highlights the importance of eNOS role in CAD. Samples were not available for additional experiments of NOS enzyme expression in PBMCs of patients with CAD. It would be advantageous to examine the NOS expression at the protein level using methods such as western blot compared to mRNA expression level in future studies to validate their role in pathogenesis of CAD.

## 6. Conclusion

 In the present study, we demonstrated that eNOS expression was increased in the CAD people compared to normal people. We also showed that eNOS intron 4b/4a 27 bp-VNTR polymorphism was associated with CAD, and aa genotype frequency was significantly higher in CAD people in comparison with normal people. We also illustrated that aa genotype carriers have increased levels of eNOS expression compared with the same genotype in normal group which might have contribution to reduced risk of CAD.

## Figures and Tables

**Table 1:** Baseline characteristics in Iranian subjects with and without CAD.

Variable	With coronary artery stenosis CAD^+^	Without coronary artery stenosis CAD^−^
Sex (male) *N* (%)	32 (60.4)	37 (68.5)
Age^*£*^ (yr)	63 ± 8.5	55 ± 11
Current smokers *N* (%)	14 (25.9)	7 (13.2)
Hypertension *N* (%)	41 (75.9)	27 (50.9)
Diabetes mellitus *N* (%)	29 (53.7)	12 (22.6)
Dyslipidemia	27 (50)	13 (24.5)
TChol^*£*^	188 ± 47	175 ± 38
TG^*£*^	188 ± 84	165 ± 80
LDL^*£*^	116 ± 41	118 ± 37
HDL^*£*^	44 ± 17	43 ± 12
Past MI *N* (%)	28 (51.9)	3 (5.7)

TChol: total cholesterol, TG: triglyceride, LDL: low density lipoprotein, HDL: high-density lipoprotein, Past MI: past history of myocardial infarction, ^*£*^variables are described based on mean ± standard deviation, *N* (number) and (%).

**Table 2:** Primer sequences for real-time PCR quantification.

Gene	Primer pair sequences	Amplicon length
*HPRT *		
F	5′-CCTGGCGTCGTGATTAGTGAT-3′	131 bp
R	5′-AGACGTTCAGTCCTGTCCATAA-3′	
*eNOS *		
F	5′-TGGTACATGAGCACTGAGATCG-3′	148 bp
R	5′-CCACGTTGATTTCCACTGCTG-3′	

**Table 3:** eNOS intron 4 VNTR genotype frequencies in CAD^+^ and CAD^−^ group.

eNos VNTR genotype	CAD^+^ *N* (%)	CAD^−^ *N* (%)
bb	28 55%	30 66%
ab	10 20%	12 26%
aa	13 25%∗	4 8%

^*^aa versus ab + bb; *P* = 0.02, OR = 3.5, 95%  CI = 0.98 to 16.2.
